# Wogonin protects against bleomycin-induced mouse pulmonary fibrosis via the inhibition of CDK9/p53-mediated cell senescence

**DOI:** 10.3389/fphar.2024.1407891

**Published:** 2024-07-08

**Authors:** Libo Wang, Fei Lin, Youli Liu, Wei Li, Qingjie Ding, Xulei Duan, Lin Yang, Zhengyu Bai, Min Zhang, Yuming Guo

**Affiliations:** ^1^ Collaborative Innovation Center of Henan Province for Green Manufacturing of Fine Chemicals, Key Laboratory of Green Chemical Media and Reactions, Ministry of Education, School of Chemistry and Chemical Engineering, Henan Normal University, Xinxiang, China; ^2^ Department of Cardiology, Life Science Research Center, The First Affiliated Hospital of Xinxiang Medical University, Xinxiang, China; ^3^ King’s College London British Heart Foundation Centre of Research Excellence, School of Cardiovascular and Metabolic Medicine and Sciences, London, United Kingdom

**Keywords:** pulmonary fibrosis, cellular senescence, wogonin, bleomycin, cyclin-dependent kinase

## Abstract

Pulmonary fibrosis (PF) is a fatal interstitial lung disease associated with declining pulmonary function but currently with few effective drugs. Cellular senescence has been implicated in the pathogenesis of PF and could be a potential therapeutic target. Emerging evidence suggests wogonin, the bioactive compound isolated from *Scutellaria baicalensis*, owns the anti-senescence properties, however, the possible impact of wogonin on PF and the potential mechanisms remain unclear. In this study, a well-established mouse model of PF was utilized which mice were administrated with bleomycin (BLM). Strikingly, wogonin treatment significantly reduced fibrosis deposition in the lung induced by BLM. *In vitro*, wogonin also suppressed fibrotic markers of cultured epithelial cells stimulated by BLM or hydrogen peroxide. Mechanistic investigation revealed that wogonin attenuated the expressions of DNA damage marker γ-H2AX and senescence-related markers including phosphorylated p53, p21, retinoblastoma protein (pRB), and senescence-associated β-galactosidase (SA-β-gal). Moreover, wogonin, as a direct and selective inhibitor of cyclin-dependent kinase 9 (CDK9), exhibited anti-fibrotic capacity by inhibiting CDK9 and p53/p21 signalling. In conclusion, wogonin protects against BLM-induced PF in mice through the inhibition of cell senescence via the regulation of CDK9/p53 and DNA damage pathway. This is the first study to demonstrate the beneficial effect of wogonin on PF, and its implication as a novel candidate for PF therapy.

## Introduction

Since December 2019, SARS coronavirus 2 (SARS-CoV-2) has caused the most severe coronavirus pandemic, with pulmonary fibrosis (PF) emerging as one of the most severe long-term complications (Wendisch et al., 2021; El-Aarag et al., 2022; Parimon et al., 2022). PF is a progressive disease in which the healthy lung anatomy undergoes complex changes, including active remodelling, extracellular matrix deposition, and dramatic alterations in the phenotype of endothelial cells, interstitial cells, and alveolar epithelial cells (Richeldi et al., 2017). Idiopathic PF (IPF) is the most common form of pulmonary fibrosis, with a median overall survival time of 4.5 years, making it one of the gravest lung diseases, second only to lung cancer (Mailleux and Crestani, 2022). Currently, there are few effective drugs and limited treatment strategies for PF (Richeldi et al., 2017). Previous studies have showed that various pharmacotherapeutic drugs, such as prednisolone, azathioprine, acetylcysteine, warfarin, nintedanib, and pirfenidone, which are currently recommended for PF patients, exhibited unfavourable side effects (Raghu et al., 2012; Vancheri et al., 2018). Therefore, in-depth exploration of PF pathogenesis and the identification of effective therapeutic strategies are urgently needed.

The pathogenesis of PF is complex. Although chronic inflammatory disorders have been implicated, anti-inflammatory therapy has not improved outcomes (Behr, 2012). Currently, PF is widely considered as a consequence of multiple interacting factors, including genetics, connective tissue diseases, infections, and environmental risk factors, alongside recurrent local micro-injuries and accelerated cell senescence in the lungs. This leads to aberrant repair of injured interstitial and alveolar cells and subsequent collagen deposition (Martinez et al., 2017; Lederer and Martinez, 2018). While the pathogenesis of PF is not fully understood, emerging evidence strongly indicates that excessive cellular senescence plays a crucial role in its development (Schafer et al., 2017; Liu and Liu, 2020). Recent single cell RNA-sequencing (scRNA-Seq) has showed that senescence and senescence-related pathways are among the top upregulated pathways in epithelial cells in IPF lung tissues, further highlighting cellular senescence acts as a key mediator of IPF pathology (Yao et al., 2021). Previous studies have revealed that inhibiting cell senescence in epithelial cells or fibroblasts in the lung can mitigate pulmonary fibrosis (Jiang et al., 2017; Bernard et al., 2020; Wan et al., 2024).

Senescence is a complex phenomenon that occurs in response to various cellular stresses, leading to proliferation arrest (Mosteiro et al., 2016). Different mediators of senescence have been identified, including senescence-associated β-galactosidase (SA-β-gal), tumor protein 53 (p53), cyclin-dependent kinase inhibitor 1 (p21), and retinoblastoma protein (pRB) (Campisi & d’Adda di Fagagna, 2007; Muñoz-Espín et al., 2013; Bieging et al., 2014; Huang et al., 2022). Particularly, the p53 protein plays a critical role in the process of cellular senescence across diverse disease settings (Rufini et al., 2013). During cellular stress, the stability of p53 is enhanced after phosphorylation by stress-induced kinases, leading to increased cell cycle arrest and cellular senescence (Günther et al., 2015; Prieto et al., 2019). The phosphorylation status of p53 controls both the activation and termination of p53-mediated transcriptional programs during different stages of the cellular DNA damage response (DDR), including the cell cycle inhibitor p21, pro-apoptotic markers, and DNA repair process (Nicolai et al., 2015).

Nuclear DNA damage, which mainly results in DNA double-strand breaks, plays an important role in causing cell senescence and triggers the DDR pathway (Di Micco et al., 2021). Therefore, understanding the mechanism of cellular senescence is a prerequisite for developing effective treatment of fibrotic lung disease by targeting p53 signalling (Amor et al., 2020; Liu and Liu, 2020). It has been reported that cyclin-dependent kinase 9 (CDK9) is a major player in phosphorylating p53 (Radhakrishnan and Gartel, 2006), however, the role of the CDK9/p53 pathway in lung fibrosis remains unclear.

Wogonin (5, 7-dihydroxy-8-methoxyflavone) is a bioactive molecule isolated from the root of Chinese herb *Scutellaria baicalensis*, traditionally used for its antioxidant, anti-senescence, anti-inflammatory, and anti-viral properties (Lucas et al., 2015; Yang et al., 2020; Banik et al., 2022). Notably, wogonin is a direct and selective inhibitor of CDK9 (Wang et al., 2019). While wogonin has demonstrated effective anti-fibrotic properties in the liver, kidney, and heart (Khan et al., 2016; Meng et al., 2016; Du et al., 2019), its effects on PF have not been fully investigated. In this study, we report that wogonin significantly mitigates PF in a bleomycin (BLM)-induced mouse lung fibrosis model *in vivo,* and suppresses fibrotic marker levels in cultured epithelial cells stimulated by BLM *in vitro*. Our research further elucidates a potential molecular mechanism of wogonin, involving CDK9-mediated p53 phosphorylation, downregulation of p21 signaling, and reduction of DNA damage and cellular senescence. Increased expression of CDK9 is also evident in human lung tissues affected by PF. These findings strongly suggest wogonin is a novel and promising candidate for PF therapy.

## Materials and methods

### Human subjects

Archived and deidentified lung tissue samples were obtained from the First Affiliated Hospital of Xinxiang Medical University Pathology Tissue Service and were surgical remnants of biopsies or lungs explanted from patients with IPF who underwent pulmonary transplantation. This study was performed according to the principles of the Declaration of Helsinki principles. The study was approved by the ethical committee of the First Affiliated Hospital of Xinxiang Medical University and informed consent was obtained before tissue acquisition when indicated.

### Experimental mouse model of lung fibrosis

All animal care and experiments were conducted in compliance with the requirements of the National Act on the Use of Experimental Animals (China) and were approved by the Institutional Animal Care and Ethics Committee of Xinxiang Medical University (No. EC-022-129). Male 9- to 12-week-old C57BL/6 mice were purchased from the Beijing Vital River Laboratory Animal Technology Co., Ltd. The mice were anesthetized with 1% isoflurane and then intratracheally administered 1.5 U/kg BLM (Macklin, Shanghai, China) in 50 μl of sterile saline. Control mice were administered the same volume of sterile saline. To test the therapeutic efficacy of wogonin in BLM-induced established fibrosis, wogonin was intraperitoneally administered at 50 mg/kg/2-day for 2 weeks and the mice were sacrificed on day 21. Equivalent volumes of saline were used as controls in all experiments.

### Hydroxyproline assays

Lung hydroxyproline content was detected using a hydroxyproline colorimetric assay kit from Nanjing Jiancheng Co., Ltd. (Nanjing, China), according to the manufacturer’s instructions, as previously described (Song et al., 2022). Data are expressed as µg of hydroxyproline in the right lung.

### Histology and immunohistochemistry

The mice were humanely euthanized and tissue samples were rapidly harvested and washed in PBS to remove blood, fixed in 4% paraformaldehyde immediately, dehydrated, embedded in paraffin wax and sectioned (4 μm) for further use. Collagen deposition was stained using Masson’s trichrome kit according to the manufacturer’s instructions (G1346, Solarbio, Beijing, China). Immunostaining for α-SMA (1:200), CDK9 (1:200), p21 (1:200) and p-p53 (1:200) was performed after paraffin removal, rehydration and blocking, following the recommendations of the manufacturer and a protocol previously described (Liu et al., 2020). Sections diluted in PBS were incubated with the primary antibody overnight at 4°C, washed 5 times with PBS. and then incubated with a secondary antibody (Jackson, 1:500) for 1 h at room temperature. The sections were counterstained with hematoxylin. The primary antibody was replaced with a non-immune serum as a negative control. The expression levels of α-SMA, CDK9, p21 and p-p53 were immune-stained with the respective antibodies, and the fractional areas were quantified using Image J software.

### Cell culture and treatment

The lung epithelial A549 and Mel-12 cell lines were purchased from the American Type Culture Collection (ATCC, Manassas, VA, United States). The cells were cultured in Dulbecco’s modified Eagle’s medium (DMEM), supplemented with 10% (v/v) fetal bovine serum (FBS) and penicillin–streptomycin at final concentrations of 100 U/mL and 100 μg/mL, respectively. Cells were cultured at 37°C in a humidified atmosphere containing 5% CO_2_. The cells were incubated with BLM (15 mU/ml) to stimulate cell fibrotic differentiation as previously described (Yu et al., 2018), or incubated with H_2_O_2_ (200 µM ) to stimulate oxidative stress-induced senescence as previously described (Mao et al., 2010). The cells were seeded in 6-well plates at a density of 2 × 105 cells/well. After 70% confluence, cells were incubated with BLM medium which contain 10% FBS for 4 h. For rescue experiments, A549 cells were cultured with wogonin at a concentration of 20 µM, or treated with AZD4573 at a concentration of 1 µM which selectively inhibits CDK9 (S8719, Selleck) (Wang et al., 2019; Ohno et al., 2022) to specifically establish the contribution of the CDK9/p53/p21 pathway. All experiments involving A549 cells were performed on cells from independent batches in triplicates or quadruplicates.

### CDK9 constructs

Lentiviruses expressing a shRNA targeting CDK9 (Gene ID: 1025), which a short hairpin sequence targeting against CDK9 (shCDK9) was GGGAGAUCAAGAUCCUUCATT, or a scramble control (shNC): TTCTCCGAACGTGTCACGT, were generated by Shanghai GeneChem Co., Ltd. (Shanghai, China). A549 cells were cultured for 48 h following infection and selected using puromycin (2 μg/mL). Western blot analysis was used to detect the effective knockdown of CDK9.

### Quantitative RT-PCR

RNA was isolated from cells treated with or without wogonin using TRIzol reagent (Life Technologies). Amplifications were performed in optical-grade 96-well plates using an Applied Biosystems QuantStudio DX machine with Fast SYBR Green Master Mix (Yeasen, Shanghai, China). The normalized fold expressions of the tested gene relative to the β-actin control were calculated based on the 2−ΔΔCt method. Oligonucleotide primers were (forward and reverse):

β-actin: AGGCCAACCGTGAAAAGATG-3′; AGAGCATAGCCCTCGTAGATGG,

α-SMA: GTGAAGAAGAGGACAGCACTG; CCCATTCCCACCATCACC,

fibronectin: TCCACAAGCGTCATGAAGAG; CTCTGAATCCTGGCATTGGT.

### Western blotting

Total tissues or cell lysates were obtained in RIPA Lysis Buffer (P0013C, Beyotime, Beijing, China) containing phosphatase inhibitor cocktail A (P108, 1 Beyotime, Beijing, China) and protease inhibitor, PMSF at 1 mM (ST506, Beyotime, Beijing, China). To quantify phosphorylated proteins, cells were washed with PBS, and lysis buffer was immediately added to protect the phosphorylation sites. Equal amounts of lysates or protein extracts were loaded onto sodium dodecyl sulfate-polyacrylamide gels, and then transferred to polyvinylidene fluoride (PVDF) membranes. After blocking with 5% no-fat milk in TBST (150 mM NaCl, 20 mM Tris, 0.05% Tween-20) at room temperature for 1-1.5 h, then the PVDF membranes were incubated with primary antibodies at 4°C overnight, followed by incubation with the appropriate horseradish peroxidase (HRP)-conjugated secondary antibodies. Protein bands were visualized using an enhanced chemiluminescence detection system on Amersham Imager 600 (GE Healthcare). Antibodies used were: CDK9 (Abcam, 76320); phospho-p53 (Abcam, 33889); fibronectin (Abcam, 2413); γ-H2AX (Abcam, 81299); α-SMA (Abcam, 11003); p21(Abcam, 109199); pRB (Proteintech, 10494-1-AP); Secondary antibodies used were: goat anti-rabbit-HRP (Jackson, 111-005-045), goat anti-mouse-HRP (Jackson, 111-005-062). Densitometric analysis was performed using Image J software (NIH, United States).

### Immunofluorescence

The lung sections or cells in 24-well plates were permeabilized with 0.25% TritonX-100 in PBS for 10 min. After blocking with 5% BSA for 1 h at room temperature, sections were incubated with CDK9 antibody (Abcam, 76320), fibronectin (Abcam, 2413) or γ-H2AX (Abcam, 81299) overnight at 4°C. After washing 5 times with PBS, cells were incubated with Alexa Fluor secondary antibodies (A0423, Beyotime, Beijing, China) (1:300 dilution) for 1 h at room temperature in the dark. Cell nuclei were counterstained with DAPI. Fluorescent images were obtained using a Zeiss fluorescence microscope.

### SA-β-Galactosidase Staining

Senescent cells were evaluated with an SA-β-galactosidase staining kit (Beyotime, Beijing, China), according to the manufacturer’s instructions. The cells were cultured in 6-well plates, with or without BLM, in triplicates. After senescence induction using BLM and wogonin treatment respectively, the cells were fixed and stained with complete β-gal Stain Solution at 37°C overnight. A549 or Mel-12 cells were visualized under a bright-field microscope at ×10 or ×20 magnification, respectively. SA-β-gal-positive cells were quantified in 4 randomly selected fields per individual sample and using ImageJ software for quantification.

### Statistics

All data in this study were presented as mean ± SEM. Comparisons of groups were performed using Student’s unpaired t-test or one or two-way ANOVA, as appropriate. A post-hoc Tukey’s test was performed to isolate differences. *p*-values < 0.05 were considered statistically significant. GraphPad Prism 9.0 statistical software was used for all the data analyses involved in this study.

## Results

### Wogonin protects the lung fibrosis in an experimental mouse model of PF *in vivo*

**FIGURE 1:**
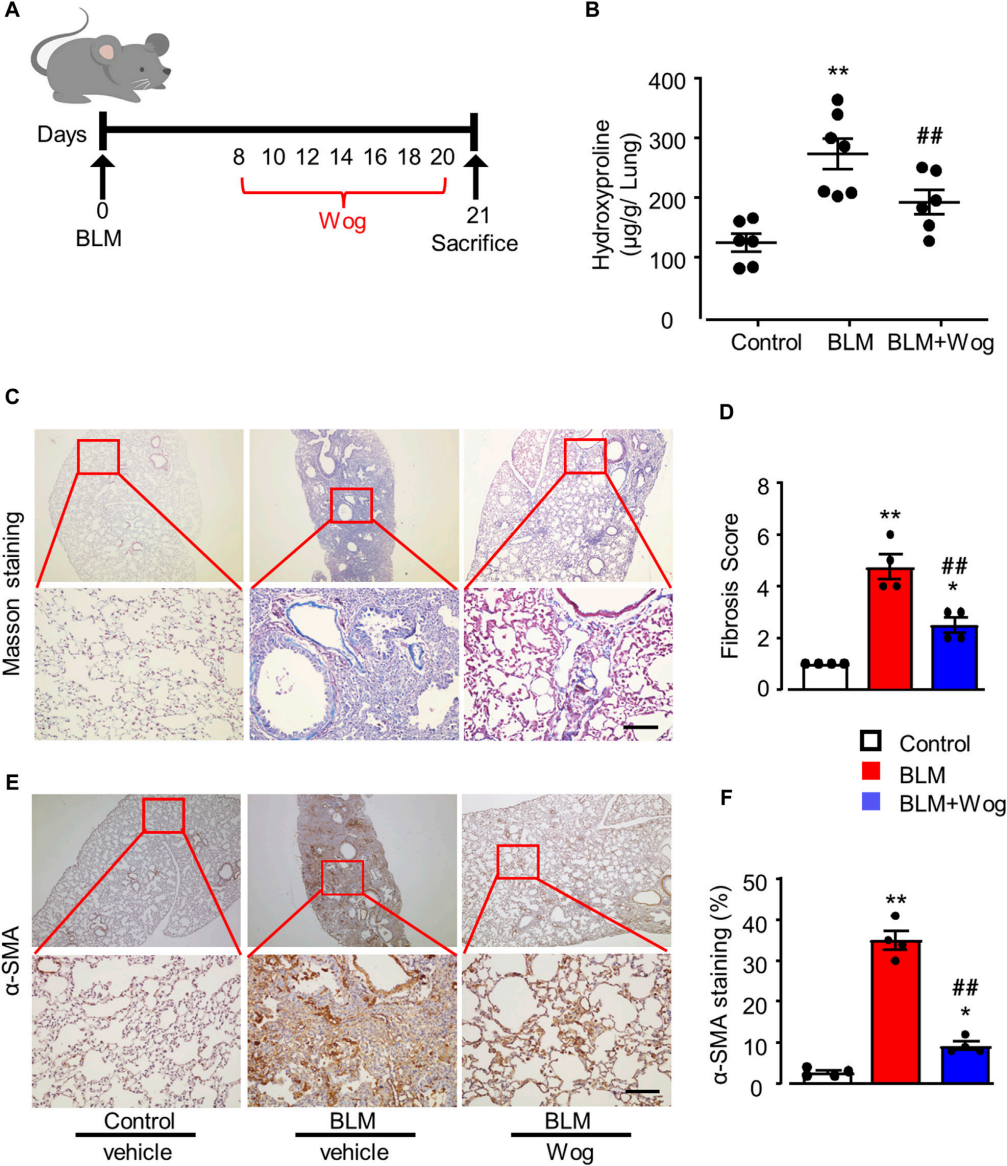
Wogonin protects the lung fibrosis in an experimental mouse model of PF *in vivo*. **(A)** Outline of the dosing design of wogonin (Wog) in 8–10-week-old C57BL/6 mice with a well-established pulmonary fibrosis (PF) model induced by bleomycin (BLM). **(B)** Hydroxyproline content in mouse lungs with 50 mg/kg wogonin treatment; n = six to seven mice/group. **(C)** Representative images of Masson’s trichrome staining and **(D)** Fibrosis scores based on stained lung sections. **(E)** immunohistochemical analysis of α-SMA in the lung sections and **(F)** Relative quantification of immunohistochemical analysis of α-SMA with or without wogonin treatment; n = 4/group; Scale bars: 200 μm; *p < 0.05, **p < 0.01, compared with control animals; ##*p* < 0.01, compared with BLM group, one-way ANOVA with a *post hoc* Tukey’s test. All data are presented as the mean ± SEM.

Firstly, to investigate the effects of wogonin on PF *in vivo*, a well-established mouse model of PF was utilized which mice were administrated with BLM at a concentration of 1.5 U/kg *via* the oropharyngeal route to induce PF (Yu et al., 2018). Wogonin was given i.p. at the concentration of 50 mg/kg/2-day for 2 weeks starting on the day 8 after BLM treatment. Equivalent volume of saline was used for the control mice (Figure 1A). Lung hydroxyproline as a surrogate of collagen deposition (Song et al., 2022), was significantly decreased in wogonin-treated group compared with the BLM group (Figure 1B). Collagen deposition was examined with Masson’s trichrome staining and demonstrated that the wogonin group had a significant lower lung fibrosis deposition compared to those in the BLM group (Figures 1C,D). Furthermore, immunohistochemical analysis showed the reduced expression of alpha smooth muscle actin (α-SMA) in mice treated with wogonin (Figures 1E,F). These results indicate that wogonin could efficiently protect against BLM-induced PF in mice.

### Wogonin attenuates bleomycin-induced epithelial cell death and fibrotic markers *in vitro*

**FIGURE 2:**
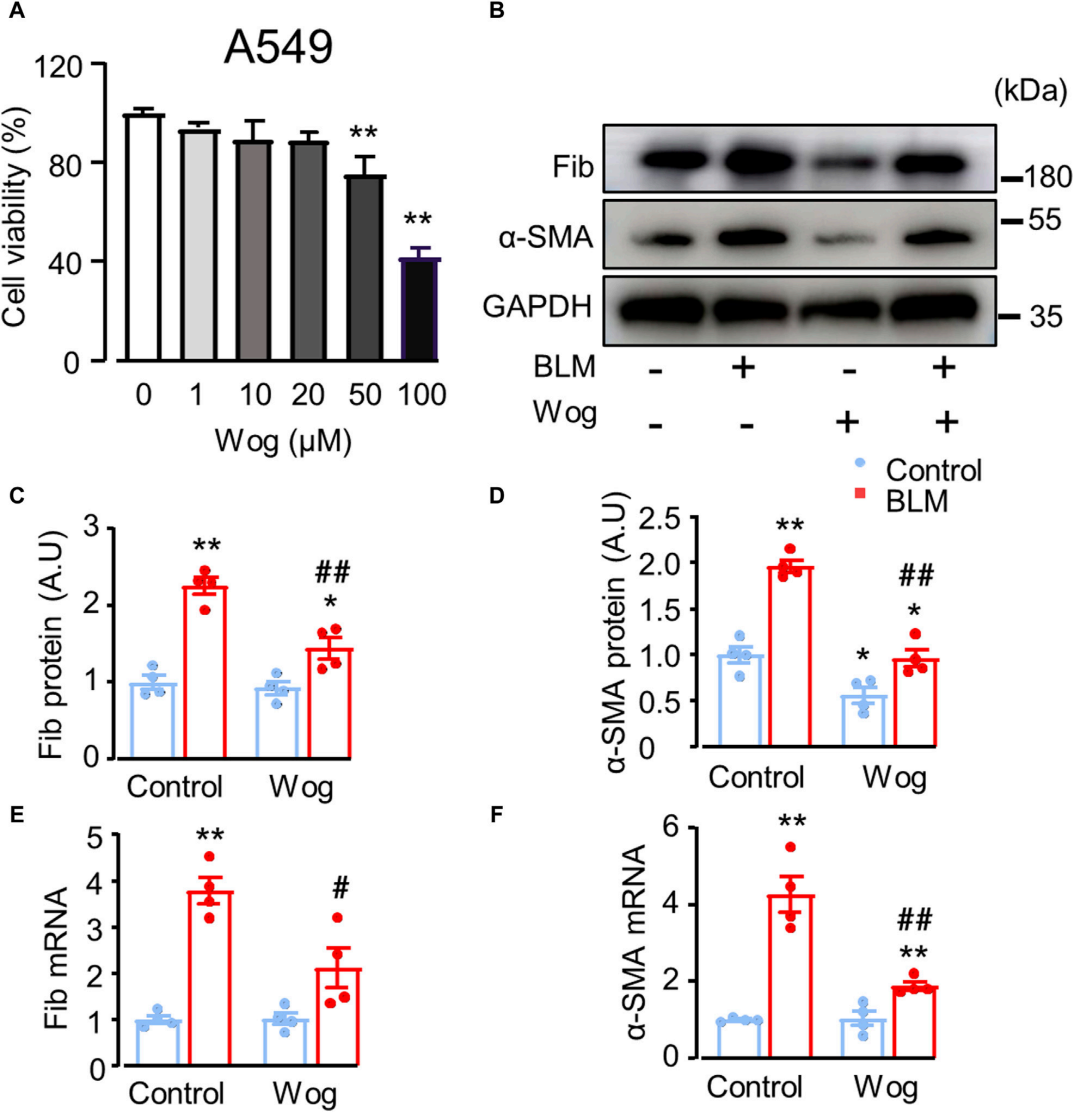
Wogonin attenuates bleomycin-induced epithelial cell death and fibrotic markers *in vitro*. **(A)** A549 cell viability by CCK-8 assay after the treatment with different concentrations of wogonin (0–100 μM) for 24 h; n = 3/group. **(B)** Western blot analysis of fibronectin (Fib) and α-SMA in A549 cells treated with or without 20 μM BLM. **(C, D)** Mean data of protein immunoblots, GAPDH as an internal control; n = 4/group. **(E, F)** mRNA levels of *Fibronectin (Fib)* and *α-SMA* by RT-qPCR; n = 4/group. *p < 0.05, **p < 0.01 compared with respective controls; #*p* < 0.05, ##*p* < 0.01 compared with BLM, 2-way ANOVA with a *post hoc* Tukey’s test. All data are presented as the mean ± SEM.

Before detecting the effect of wogonin on fibrosis at cellular level *in vitro*, we examined whether wogonin was toxic to lung epithelial A549 cells. The results of CCK8 assay demonstrated that wogonin at the concentrations of 1–20 μM had no significant effects on cell viability (Figure 2A). Therefore, the further experiments of wogonin were conducted at the concentration of 20 μM. Next, to investigate the effect of wogonin on BLM-induced fibrotic markers *in vitro*, A549 cells were cultured and stimulated with 15 mU/mL BLM at 37°C for 4 hs (Yu et al., 2018). The BLM-medium was then replaced with wogonin for 24 h. Western blot analysis showed that treatment of BLM resulted in significant increases in the protein levels of two fibrotic markers: fibronectin (Fib) and α-SMA, the effect was markedly attenuated by wogonin (Figures 2B–D). RT-qPCR similarly revealed that wogonin treatment inhibited the expression of *fibronectin* and *α-SMA* compared with BLM groups, indicating the capacity of wogonin to inhibit the fibrotic phenotype of epithelial cells *in vitro* (Figures 2E,F).

### Wogonin attenuates BLM-induced fibrotic markers by alleviating cell senescence via the inhibition of p53 phosphorylation

**FIGURE 3:**
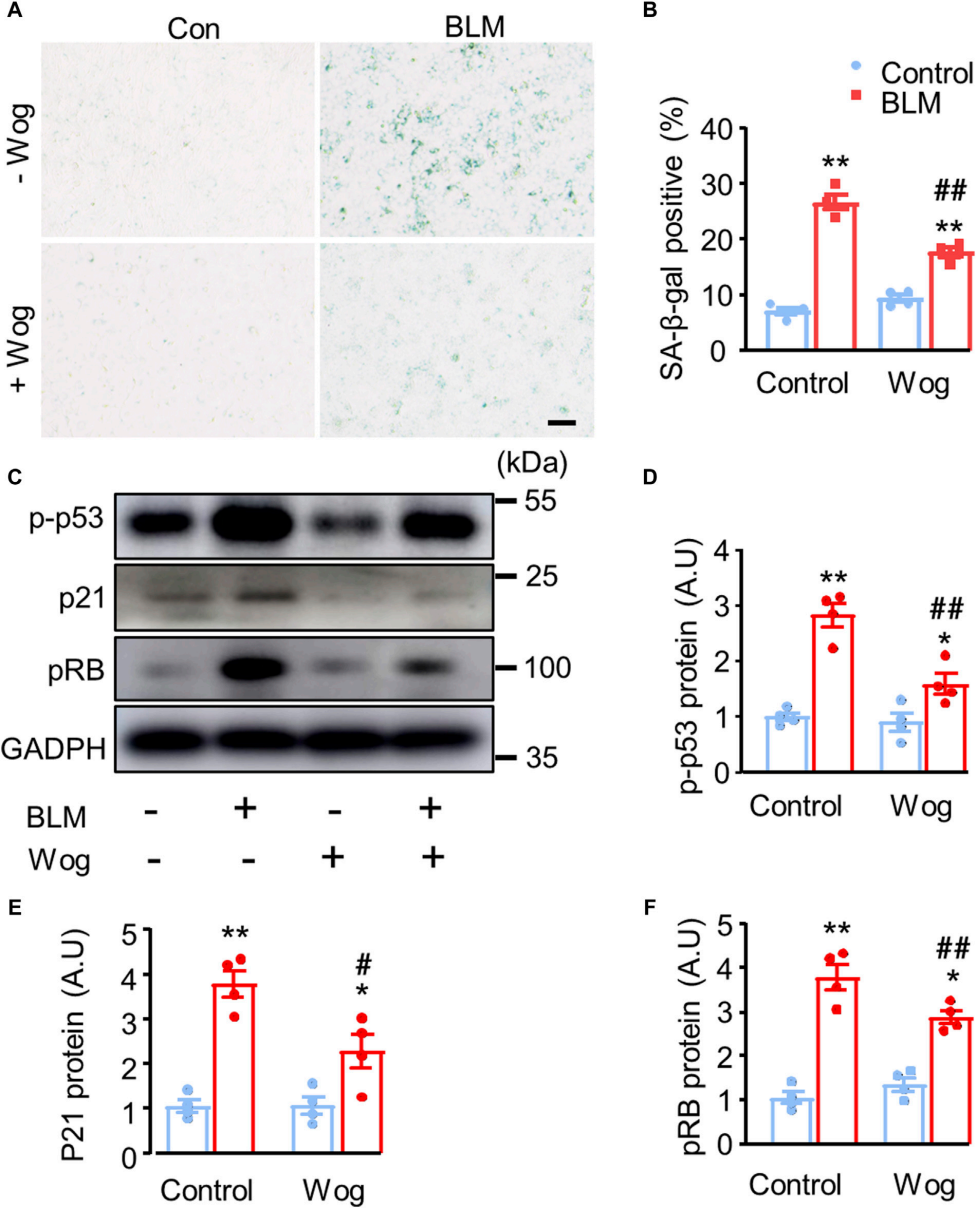
Wogonin attenuates BLM-induced fibrotic markers by alleviating cell senescence via the inhibition of p53 phosphorylation. **(A)** SA-β-gal staining and **(B)** quantification of SA-β-gal-positive cells. scale bars: 100 μm. **(C)** Protein levels of senescence markers p-p53, p21 and pRB by Western blot analysis. **(D–F)** Mean data of protein immunoblots; n = 4/group. *p < 0.05, **p < 0.01 compared with respective controls; #*p* < 0.05, ##*p* < 0.01, compared with BLM; 2-way ANOVA with a *post hoc* Tukey’s test. All data are presented as the mean ± SEM.

It has been reported that the activation of cellular senescence signalling promotes lung fibrosis formation in mice (Kuwano et al., 1996; Liu and Liu, 2020). Thus, we next explored whether the senescence pathway was involved in PF and whether wogonin could suppress it. SA-β-gal activity, a widely used marker of senescent cells, was markedly increased in BLM-stimulated A549 cells *in vitro* (Debacq-Chainiaux et al., 2009). Importantly, BLM-induced activation of SA-β-gal was significantly diminished by wogonin treatment (positive area %; 26.7 ± 1.3 vs. 17.7 ± 0.8) (Figures 3A,B). Western blot analysis demonstrated that BLM increased p53 phosphorylation (p-p53) with concomitant upregulation of p21 and pRB, whereas wogonin treatment significantly suppressed the levels of p-p53, p21, and pRB (Figures 3C–F). These results suggest that wogonin attenuated BLM-induced fibrotic markers in epithelial A549 cells by inhibiting cellular senescence through p53/p21 signalling, at least in part, by decreasing p53 phosphorylation.

### Wogonin attenuates oxidative stress-induced cell senescence via inhibiting the DNA damage

**FIGURE 4:**
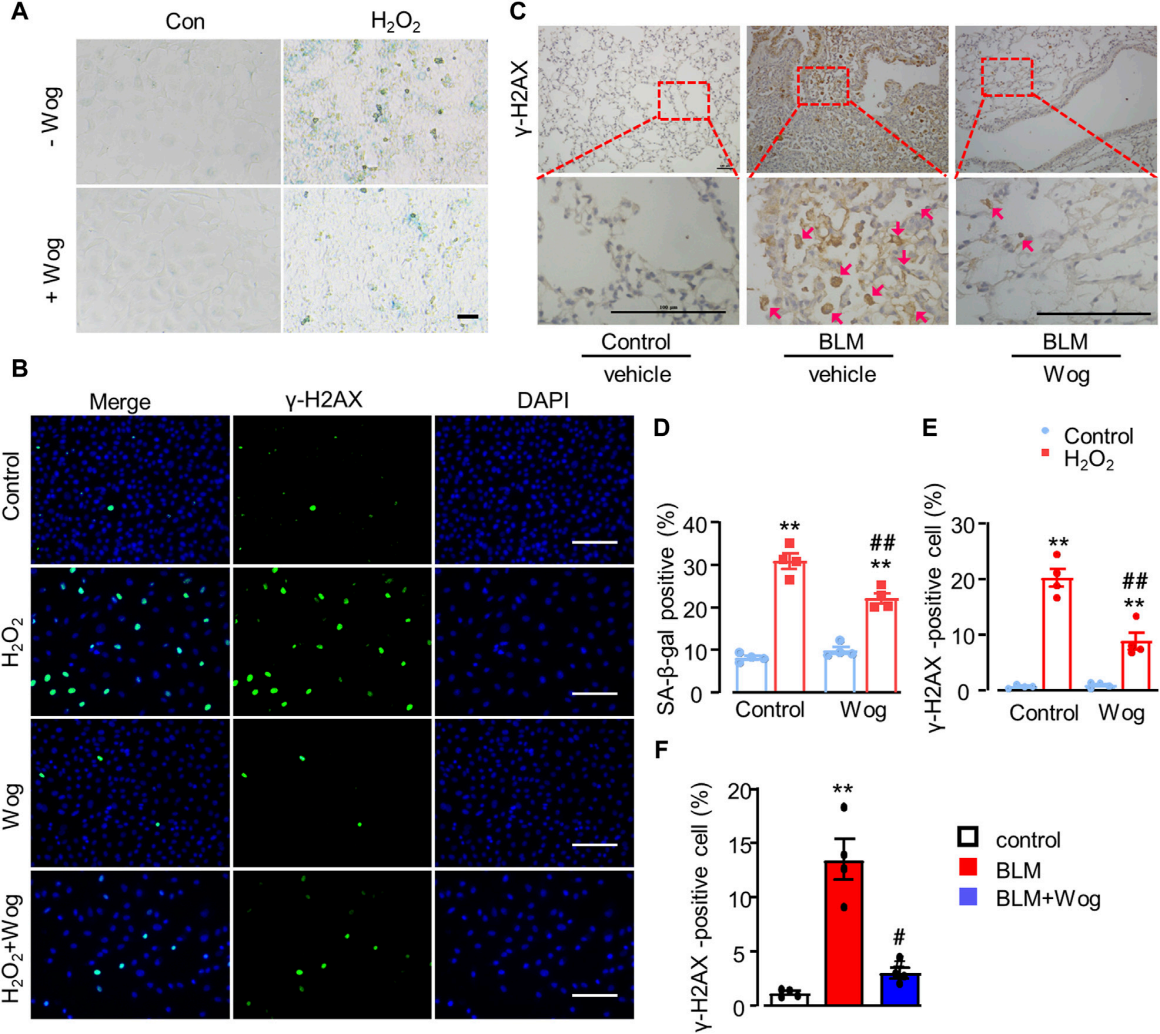
Wogonin attenuates oxidative stress-induced senescence by alleviating DNA damage. **(A)** SA-β-gal staining and **(D)** quantification of SA-β-gal-positive after H_2_O_2_ (200 μM) stimulation with or without wogonin treament. scale bars: 100 μm. **(B)** Immunofluorescence staining of γ-H2AX in epithelial A549 cells and **(E)** quantification of γ-H2AX positive cells. Scale bar: 100 μm. **(C)** Representative immunohistochemical staining of γ-H2AX in the mouse lung sections with or without wogonin treatment and **(F)** relative quantification for γ-H2AX; n = 4/group; **p < 0.01 compared with respective controls; #*p* < 0.05, ##*p* < 0.01, compared with BLM; 2-way ANOVA with a *post hoc* Tukey’s test. All data are presented as the mean ± SEM.

Oxidative stress critically contributes to pulmonary fibrosis (Otoupalova et al., 2005). H_2_O_2_ is widely used as a well-established inducer of DNA damage and cell senescence. Thus, we treated A549 cells with H_2_O_2_ at the concentration of 200 μM with or without wogonin, then examined senescence as well as expressions of DNA damage related markers. As expected, H_2_O_2_ induced A549 cell senescence as revealed by the increased expression of SA-β-gal, the effect was significantly attenuated by wogonin treatment (positive area %; 30.97 ± 1.79 vs. 22.17 ± 1.22) (Figures 4A,D). DNA damage plays an important role in triggering senescence (Zhao et al., 2023). In order to verify the level of DNA damage, we applied immunofluorescence to detect the expression of DNA damage marker γ-H2AX in A549 cells after H_2_O_2_ stimulation with or without wogonin. We found that H_2_O_2_ increased γ-H2AX expression in nucleus while wogonin significantly decreased γ-H2AX expression (Figures 4B,E). The attenuation of γ-H2AX by wogonin was further confirmed by immunohistochemical analysis *in vivo* in mice treated with BLM (Figures 4C,F). Moreover, the beneficial effects of wogonin against DNA damage, cellular senescence and fibrosis *in vitro* was replicated by the use of another mouse lung epithelial cell line Mel-12. CCK8 assay demonstrated that wogonin at the concentrations of 1–20 μM had no significant effects on cell viability in Mel-12 cells (Supplementary Figure S1A). SA-β-gal activity was significantly attenuated by the treatment with wogonin in Mel-12 compared with BLM alone (Supplementary Figure S1B,D). Immunofluorescence also showed the expression of γ-H2AX was significantly decreased in wogonin group compared with BLM group (Supplementary Figure S1C,E). These results support that wogonin attenuated BLM-induced cell senescence via the inhibition of DNA damage.

### CDK9 knockdown suppresses BLM-induced fibrotic marker levels *in vitro*

**FIGURE 5:**
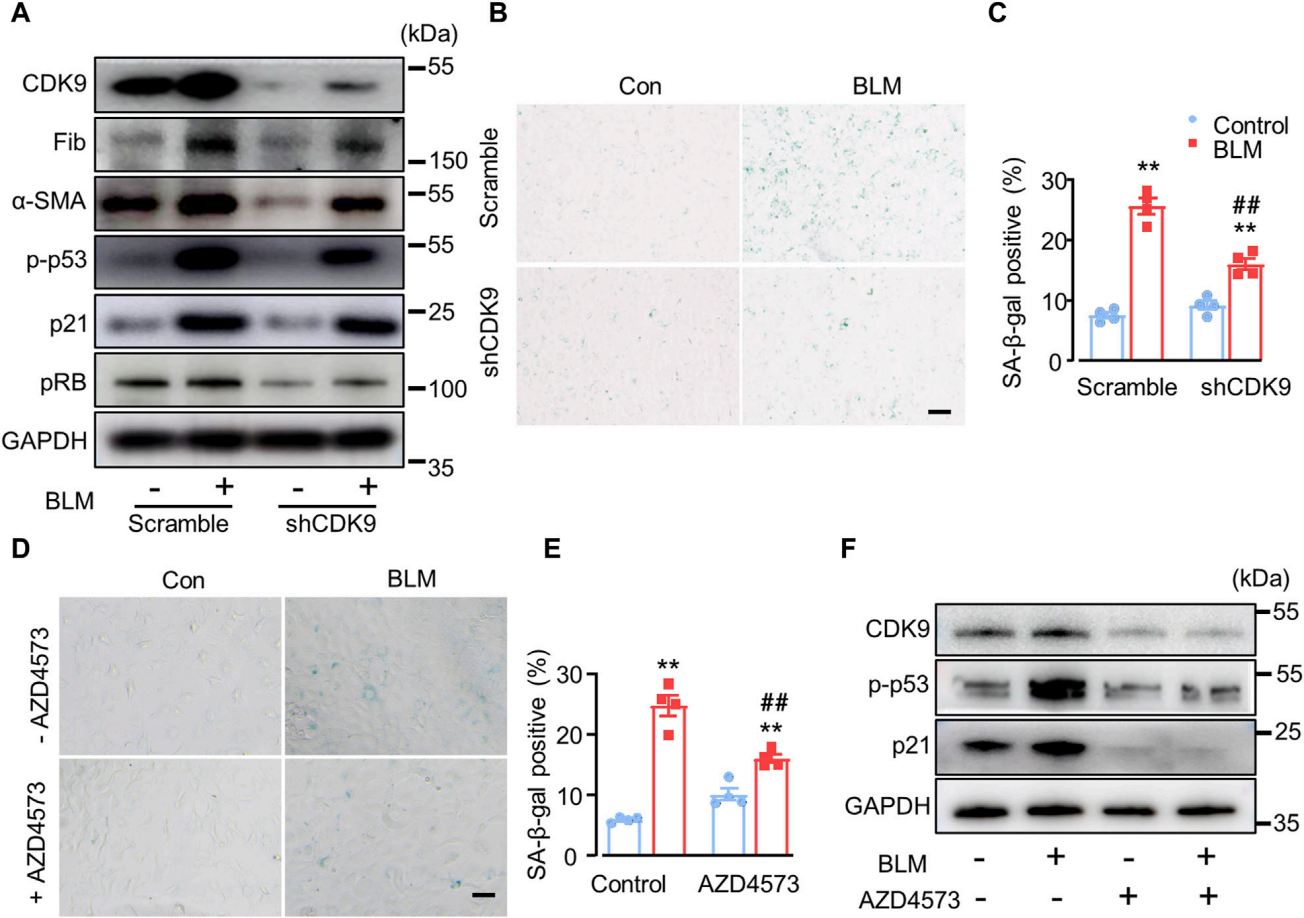
CDK9 knockdown suppresses BLM-induced fibrotic marker levels *in vitro*. **(A)** Protein levels of CDK9, fibrotic markers and senescent markers by Western blots after knockdown of CDK9 with shCDK9 lentivirus in epithelial A549 cells. n = 4/group. **(B)** SA-β-gal activity staining with shCDK9 lentivirus transfection and **(C)** the quantification of SA-β-gal-positive cells. Scale bar: 100 μm. **(D)** SA-β-gal activity staining with CDK9 inhibitor AZD4573 treatment (1 µM) and **(E)** the quantification of SA-β-gal-positive cells. Scale bar: 100 μm. **(F)** Western blot analysis of CDK9, p-p53 and p21 in epithelial A549 cells. **p < 0.01 compared with respective controls; ##*p* < 0.01, compared with BLM; 2-way ANOVA with a *post hoc* Tukey’s test. All data are presented as the mean ± SEM.

It was reported that CDK9 is an upstream activator to phosphorylate p53 (Radhakrishnan and Gartel, 2006), and wogonin is a selective inhibitor of CDK9 (Wang et al., 2019). In order to verify whether wogonin protects BLM-induced fibrosis through the inhibition of CDK9, CDK9 was knocked down in A549 cells with a short hairpin RNA (shRNA) specifically targeting its mRNA. Western blot analysis confirmed successful knockdown with a clear decrease in the protein levels of CDK9 compared to that in cells transfected with the control vector (Figure 5A; Supplementary Figure S2). Moreover, CDK9 knockdown significantly abrogated the increase of fibronectin and α-SMA compared to that in cells induced with BLM. Intriguingly, knockdown of CDK9 significantly decreased p53 phosphorylation, with or without BLM stimulation (Figure 5A; Supplementary Figure S2, suggesting that CDK9 might regulate p53 phosphorylation to a significant level. Furthermore, in contrast to the scrambled control group, knockdown of CDK9 significantly decreased p21 and pRB expression (Figure 5A; Supplementary Figure S2). Increased SA-β-gal activity staining was observed in BLM-induced cells compared to the control group but was suppressed after CDK9 knockdown (Figures 5B,C). AZD4573, a selective CDK9 inhibitor, was used to further confirm the role of CDK9 in wogonin-mediated protection. A549 cells were cultured and stimulated with 15 mU/mL BLM for 4 h (Yu et al., 2018). The BLM-medium was then replaced with AZD4573 for 24 h. SA-β-gal activity staining was obviously reduced in cells treated with AZD4573 compared to the BLM group (Figures 5D,E). Western blot analysis showed that treatment of AZD4573 resulted in significant decreases in the protein levels of CDK9 and senescence markers p-p53 and p21 (Figure 5F). Thus, wogonin exhibited anti-fibrotic capacity by inhibiting CDK9-mediated p53/p21 signalling, and overall, CDK9 knockdown/inhibition and wogonin treatment had comparable effects.

### Wogonin suppresses BLM-induced senescence via the inhibition of CDK9/p53 pathway *in vivo*

**FIGURE 6:**
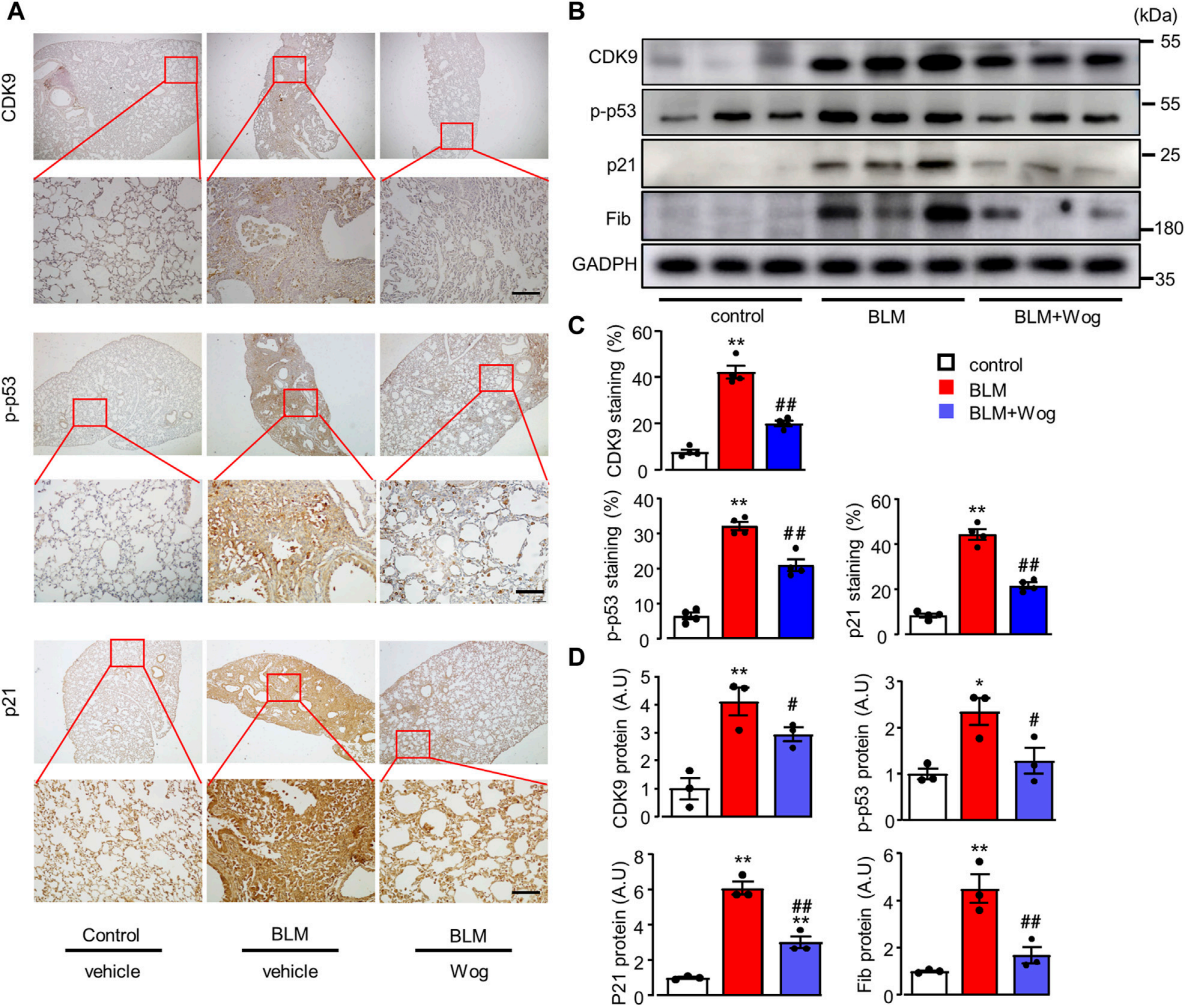
Wogonin suppresses BLM-induced senescence via the inhibition of CDK9/p53 pathway *in vivo*. **(A)** Representative immunohistochemical staining of CDK9, p-p53 and p21. Scale bars: 100 μm, and **(C)** relative quantifications of staining; n = 4/group. **(B)** Western blot analysis of CDK9, p-p53, p21, and pRB in whole lung tissue lysates and **(D)** Mean data of protein immunoblots; n = 3/group; *p < 0.05, **p < 0.01, compared with control animals; #*p* < 0.05, ##*p* < 0.01, compared with BLM group, 1-way ANOVA with a *post hoc* Tukey’s test. All data are presented as the mean ± SEM.

We further explored the potential mechanism by which wogonin attenuates cellular senescence in BLM-induced PF *in vivo*. Immunohistochemical analysis showed that CDK9, p-p53 and p21 were upregulated in the lungs of BLM-induced PF groups, whereas wogonin treatment considerably diminished the elevations of CDK9, p-p53 and p21 (Figures 6A,C). Western blot analysis further demonstrated that BLM significantly elevated CDK9, along with enhanced expression of p-p53, p21, and Fib, indicating the activation of CDK9/p53 signalling pathway in lung lysates of BLM-induced PF mice (Figures 6B,D). Importantly, wogonin significantly decreased the expression of CDK9 and the levels of p-p53 and p21, supporting that wogonin suppressed BLM-induced lung fibrosis by inhibiting CDK9/p53-mediated senescence *in vivo*.

### Elevation of CDK9 expression in the lungs of idiopathic PF patients

**FIGURE 7:**
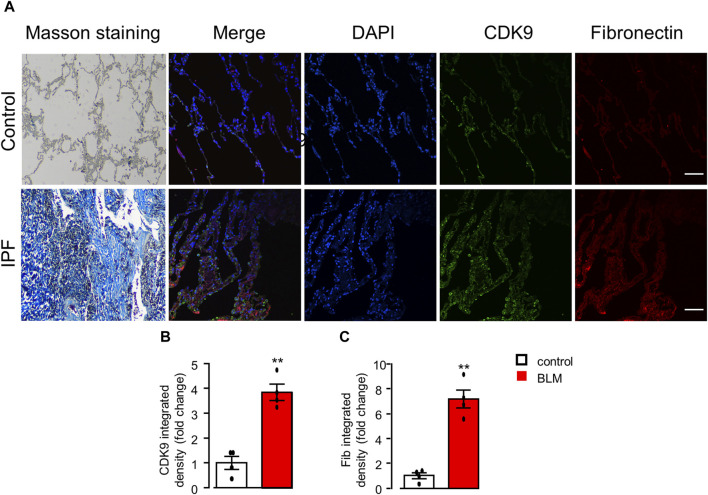
CDK9 upregulation in the lungs of PF patients. **(A)** Representative histological staining assessed to detect collagen deposition with Masson’s trichrome staining and immunofluorescence staining analysis of the expression of CDK9 and fibronectin in human lungs with or without PF. Scale bars: 100 μm. **(B, C)** Quantification of CDK9 and fibronectin with immunofluorescence integrated density (fold change). **p < 0.01 compared with controls, n = 4/group, unpaired *t*-test. All data are presented as the mean ± SEM.

In line with the upregulation of CDK9 proteins by Western blots (Figures 6B,D), the expression of CDK9 was significantly higher evaluated by immunostaining in the BLM model of lung fibrosis in mice (Figures 6A,C). Finally, to verify the increased expression of CDK9 in human lung tissues with IPF, we used immunofluorescence to detect the expression of CDK9 and fibronectin in lung samples from normal and IPF lung tissue sections. Indeed, the expressions of CDK9 and fibronectin were significantly upregulated in human fibrotic lung tissues (Figures 7A–C).

## Discussion

In this study, we found that wogonin treatment significantly suppressed BLM-induced fibrosis both *in vitro* and *in vivo*. We also uncovered a potential mechanism underlying the protective effects of wogonin against PF. Wogonin attenuated cellular senescence by inhibiting CDK9 and DNA damage signalling pathway, leading to the inhibition of p53 phosphorylation and reductions in the expression of γ-H2AX, p21 and pRB. Other studies have reported that wogonin treatment enhances anti-fibrotic activity in other organs, such as the liver and kidneys (Zheng et al., 2020; Liu et al., 2022). Additionally, wogonin has showed anti-fibrotic effects in streptozotocin-induced diabetic myocardial fibrosis in mice by inhibiting inflammation (Khan et al., 2016). However, to our best knowledge, no studies have investigated the regulatory characteristics of wogonin in PF. Our current findings demonstrate that wogonin is a promising therapeutic candidate for BLM-induced PF in mice, and that CDK9 might be a viable target for attenuating PF.

Cellular senescence crucially contributes to fibrotic lung disease, making it an attractive therapeutic target for improving pulmonary function and physical health (Lehmann et al., 2017; Schafer et al., 2017). Thus, developing new senolytic drugs that effectively target cellular senescence could be a potential strategy to inhibit the senescence process and suppress diseases with senescence-related phenotypes (Schafer et al., 2017; Guan et al., 2022). Here, we demonstrated that the senescence-associated secretory phenotype (SASP) is related to PF both *in vivo* and *in vitro*. Previous studies have established BLM-induced PF mouse models through intratracheal injection of BLM in both wild-type and p53-deficient mice. The results indicated that inhibiting p53 expression could slow the progression of PF (Nagaraja et al., 2018). Moreover, PF patients exhibit characteristics of senescence, including upregulation of senescence-related DNA damage and elevated transcription of SASP components p16, p21, and pRB (Schafer et al., 2017). Many studies have shown that the p53/p21 pathway is crucial in regulating cell senescence during the onset and development of PF (Garner and Raj, 2008; Elyada et al., 2011). Given the positive correlation between CDK9 activity and its substrate p53, an important marker for the severity of cellular senescence (Liu et al., 2022; Liu and Gu, 2022), the CDK9/p53 pathway could be critically implicated in PF (Barnes et al., 2019). In this study, we found that CDK9 was significantly upregulated in the lung tissues of experimental mouse PF models and IPF patients, suggesting CDK9 as a potential target for treating PF.

Understanding the mechanisms of cellular senescence in PF is crucial for developing treatment for fibrotic lung disease. During cellular stress, the stability of p53 is enhanced after phosphorylation by stress-induced kinases, leading to an increase in cell cycle arrest and cellular senescence (Günther et al., 2015; Prieto et al., 2019). Thus, inhibiting p53 phosphorylation might be an effective approach to treating fibrotic lung disease (Amor et al., 2020; Liu and Liu, 2020). Several protein kinases, including CDK9, may phosphorylate p53 at different residues of the amino acid (Radhakrishnan and Gartel, 2006). Wogonin has been shown to exert significant anti-cancer activity, alone or in combination with other therapies in leukemia, possibly through CDK9-mediated p53 phosphorylation (Ball and Abdel-Wahab, 2018). Further research is needed to understand how CDK9 differentially regulates p53 phosphorylation in PF. Our results indicate that p53 phosphorylation coincided with CDK9 expression, and the alleviation of cellular senescence could be attributed to inhibiting CDK9, as the expression of senescence markers, including p21 and pRB, dramatically decreased upon CDK9 knockdown. Thus, the CDK9/p53-mediated senescence pathway could serve as a novel potential molecular target for treating PF *in vivo*.

**FIGURE 8:**
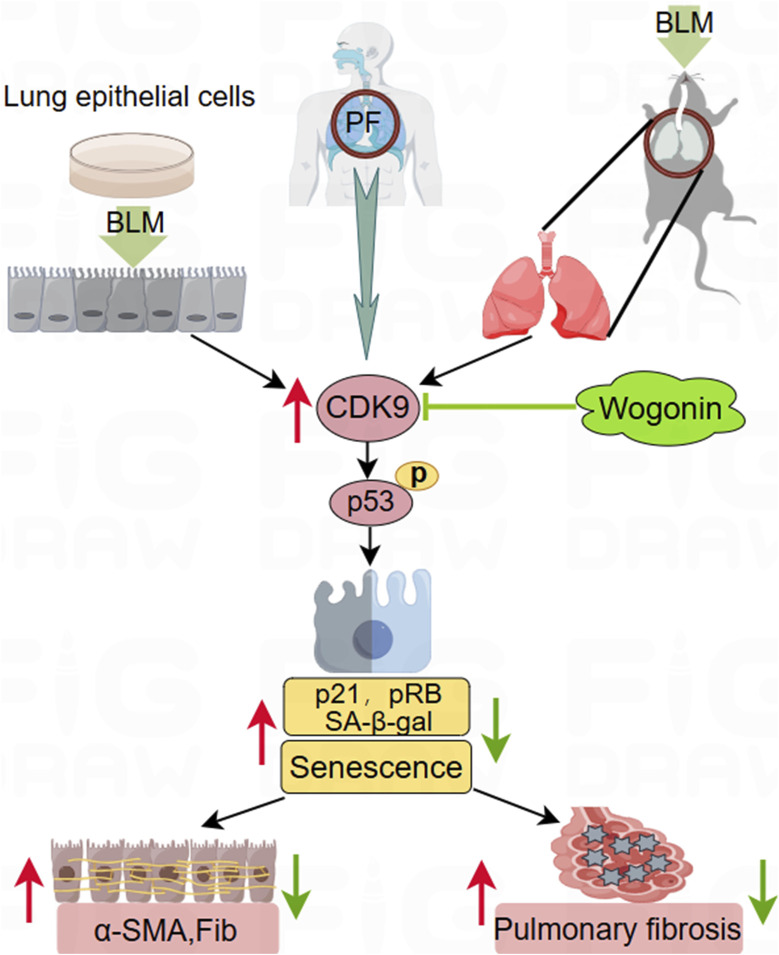
Schematic to illustrate the protection of wogonin against pulmonary fibrosis. Wogonin protects against pulmonary fibrosis (PF) in a bleomycin (BLM)-induced experimental mouse model *in vivo* and suppresses fibrotic markers in cultured epithelial cells stimulated by BLM *in vitro*. This beneficial effect is achieved by inhibiting cellular senescence through the suppression of CDK9-mediated p53 phosphorylation and downregulating p21 signalling. The upregulation of CDK9 in the lung tissues of PF patients highlights its potential as a promising therapeutic target for treating PF.

In this study, Wogonin-mediated inhibition of the CDK9/p53 signalling pathway was observed in a BLM-induced PF mouse model. Collagen deposition was assessed using Masson’s trichrome staining, and immunohistochemical analysis with anti-p21 and anti-p-p53 antibodies confirmed that wogonin inhibited senescence. These results highlight wogonin’s effect in delaying PF progression by inhibiting cellular senescence and reveal the critical role of the p53/p21 pathway in its action (Figure 8).

Nuclear DNA damage is a significant cause of cell senescence, mainly resulting in DNA double-strand breaks and triggering the DDR pathway (Di Micco et al., 2021). IPF, a progressive and terminal lung disease associated with aging, is characterized by excessive oxidative stress, contributing to DNA damage and cellular senescence (Otoupalova et al., 2005). Research has showed that the complex containing of RNA polymerase II and CDK9 is involved in DDR pathway (Pessina et al., 2019). Wogonin has been reported to alleviate DNA damage induced by air pollutant cytotoxicity in human airway epithelial cells (Ni et al., 2023). In this research, we found that wogonin attenuated BLM-induced cell senescence by inhibiting DNA damage.

There are some limitations to this study. First, antifibrotic drugs such as pirfenidone or nintedanib were not included as positive controls due to their lack of CDK9 specificity (Cheng et al., 2022). Second, the BLM-induced experimental mouse PF model differs from IPF. Third, the causal effect of wogonin on CDK9/p53 pathway in PF *in vivo* should be further investigated using gene-modified mouse models.

## Conclusion

Wogonin effectively mitigates BLM-induced PF in mice by inhibiting cellular senescence through the regulation of CDK9-mediated p53/p21 pathway. Our data support the concept that CDK9 functions as a positive regulator of cellular senescence in PF and may serve as a potential therapeutic target. Given that there is no effective strategy to prevent the progression of PF other than lung transplantation for patients with advanced disease, our findings provide new insights into the critical role of wogonin in PF. Importantly, the involvement of CDK9 in the senescence pathway presents a promising therapeutic target for treating lung fibrosis.

## Data Availability

The original contributions presented in the study are included in the article/Supplementary Material, further inquiries can be directed to the corresponding authors.

## Supplementary material

SUPPLEMENTARY FIGURE S1 Wogonin attenuates BLM-induced cell senescence and DNA damage in Mel-12 cells. **(A)** Mouse epithelial Mel-12 cells were treated with different concentrations of wogonin (0–100 μM) for 24 h. The effect of wogonin on cell viability was assayed with CCK-8 kit; n = 3/group. **(B)** SA-β-gal activity staining and **(D)** the quantification of SA-β-gal-positive cells in Mel-12 cells. Scale bar: 100 μm. **(C)** Immunofluorescence staining analysis of the expression of γ-H2AX in epithelial Mel-12 cells with or without wogonin treatment and **(E)** Quantification of γ-H2AX positive cells; n = 4/group; Scale bars, 100 μm; **p < 0.01, compared with respective control animals; ##p < 0.01, compared with BLM group, 2-way ANOVA with a *post hoc* Tukey’s test. All data are presented as the mean ± SEM.

SUPPLEMENTARY FIGURE S2 Wogonin suppresses BLM-induced senescence via the inhibition of CDK9/p53 pathway *in vivo*. Mean data of protein levels detected by Western blot analysis in Figure 5A in A549 cells treated with or without wogonin; n = 3–4/group. *p < 0.05, **p < 0.01, compared with respective control groups; #p < 0.05, ##p < 0.01, compared with BLM group, †p < 0.05, ††p < 0.01 compared with scramble or control alone, 2-way ANOVA with a *post hoc* Tukey’s test. All data are presented as the mean ± SEM.

## Abbreviations

A549, human non-small cell lung cancer cell; SARS-CoV-2, SARS coronavirus 2; PF, pulmonary fibrosis; IPF, idiopathic pulmonary fibrosis; CDK9, cyclin-dependent kinase 9; p21, Cyclin-dependent kinase inhibitor 1A; pRB, retinoblastoma protein; p53, tumor protein 53; α-SMA, alpha smooth muscle Actin; SA-β-galactosidase activity, senescence-associated β-galactosidase activity; Fib, Fibronectin; GADPH, glyceraldehyde-3-phosphate dehydrogenase; bleomycin, BLM; wogonin, Wog; Gamma H2A histone family member X, γ-H2AX.
