# Supplementary figures and images for: Wogonin protects against bleomycin-induced mouse pulmonary fibrosis via the inhibition of CDK9/p53-mediated cell senescence
Source: Front Pharmacol. 2024 Jul 8;15:1407891. doi: 10.3389/fphar.2024.1407891 (PMC11260675; doi:10.3389/fphar.2024.1407891)

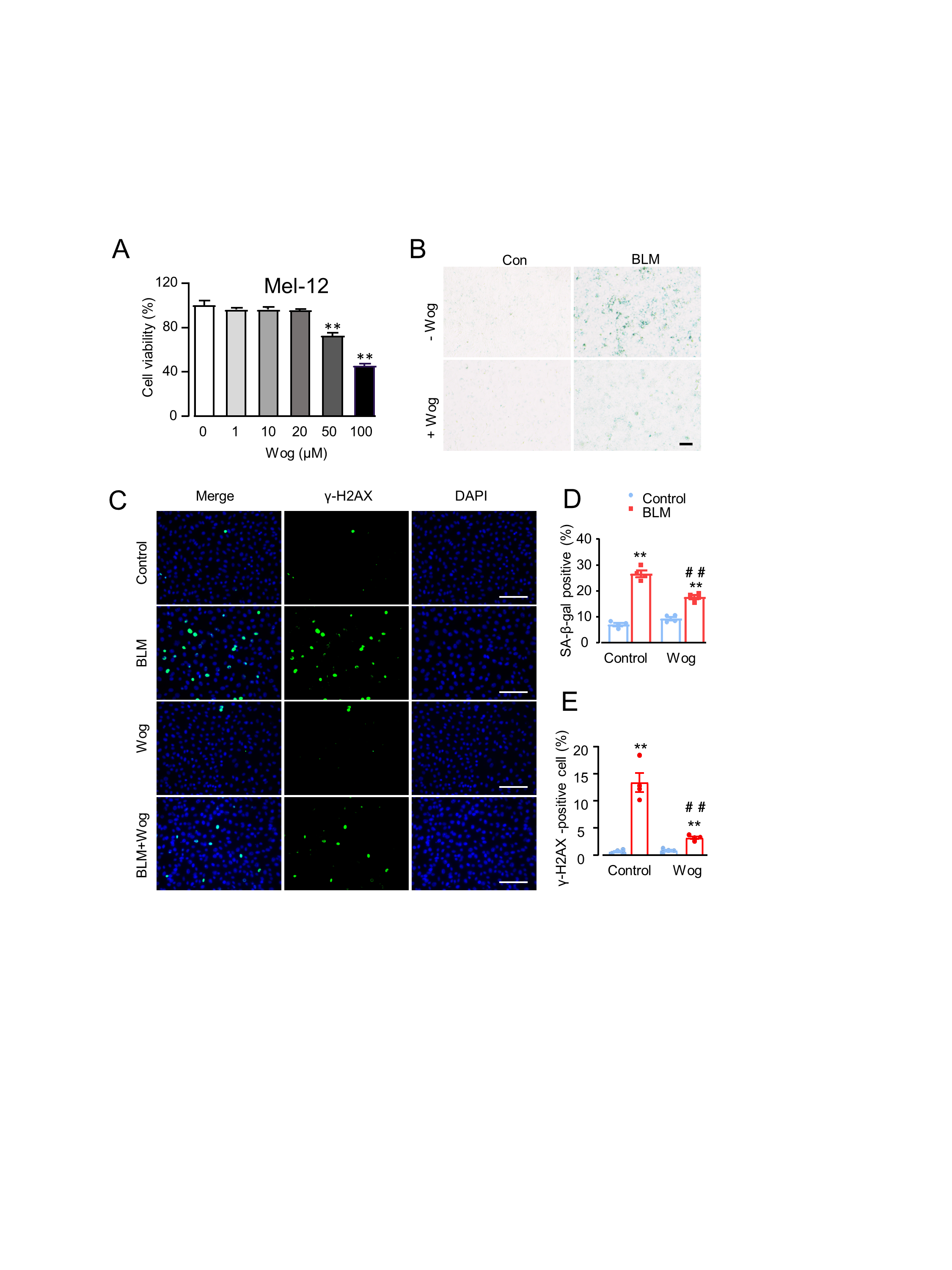

Supplement: Supplementary file 1 [file Image2.TIF]

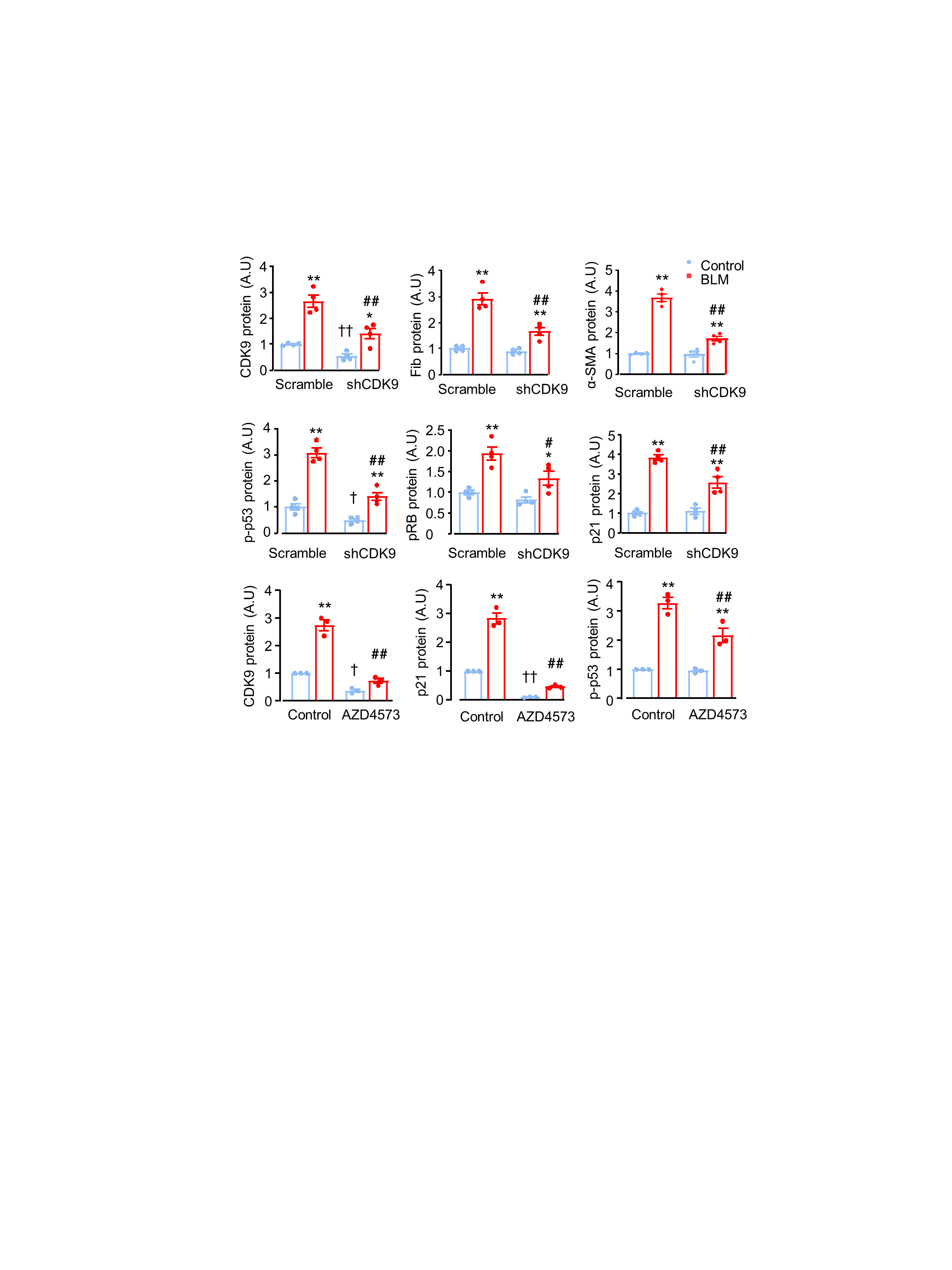

Supplement: Supplementary file 2 [file Image1.TIF]
