# Supplementary material for: Wogonin protects against bleomycin-induced mouse pulmonary fibrosis via the inhibition of CDK9/p53-mediated cell senescence
Source: Front Pharmacol. 2024 Jul 8;15:1407891. doi: 10.3389/fphar.2024.1407891 (PMC11260675; doi:10.3389/fphar.2024.1407891)

Uncut gels for Figure 2 B.

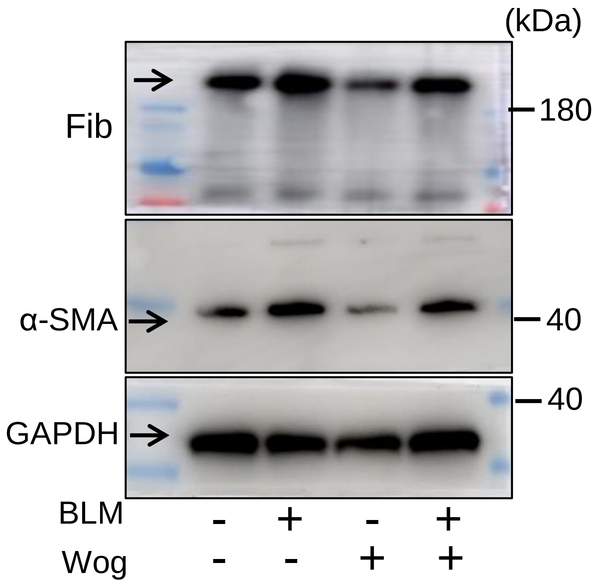

Uncut gels for Figure 3 C.

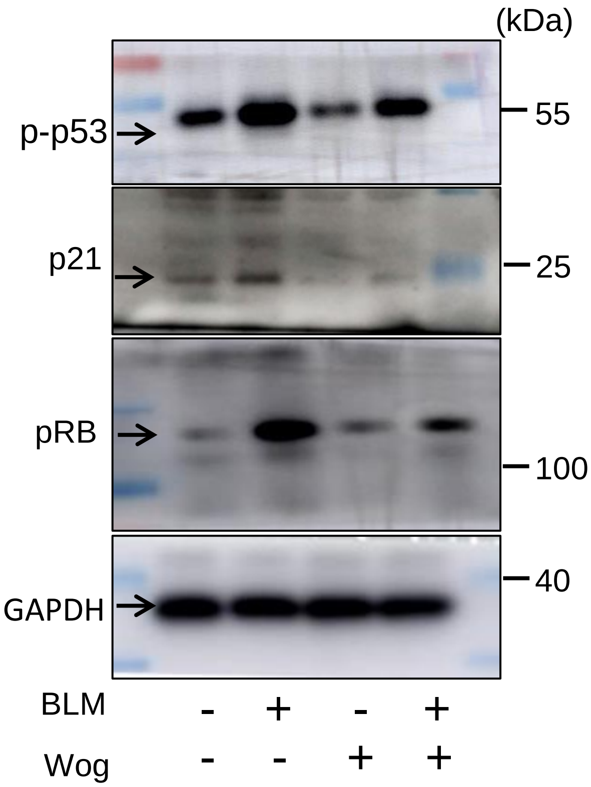

Uncut gels for Figure 5 A.

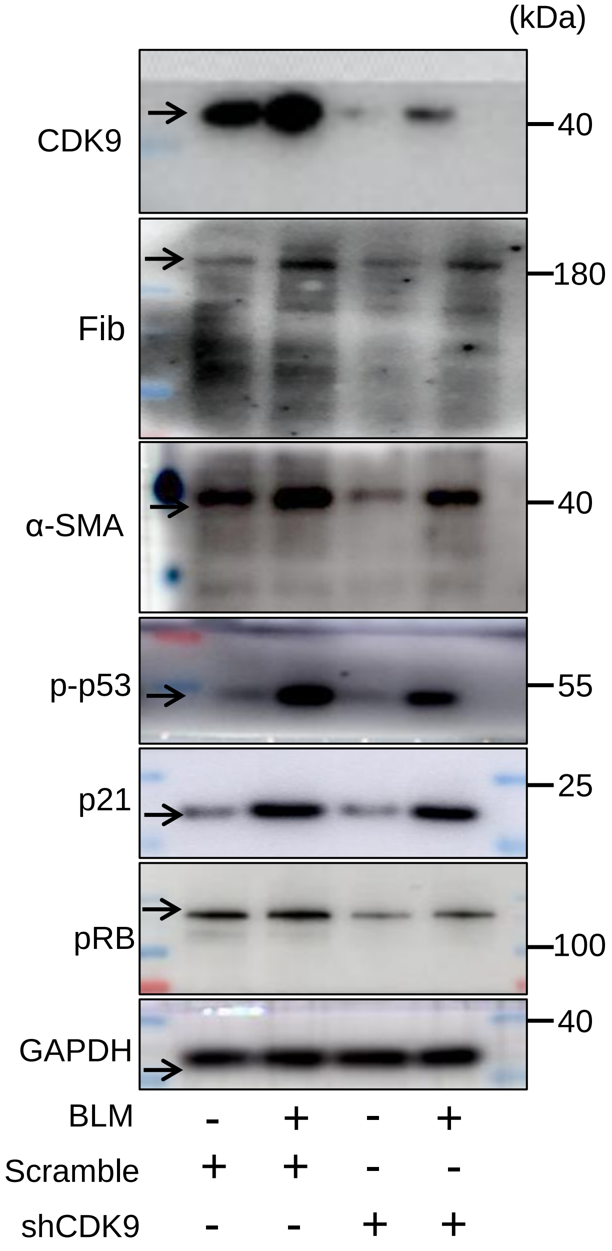

Uncut gels for Figure 5 F.

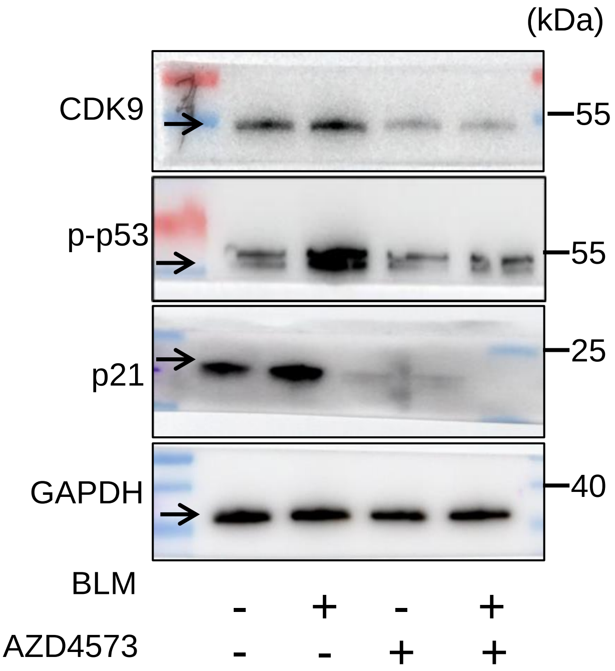

Uncut gels for Figure 6 B.

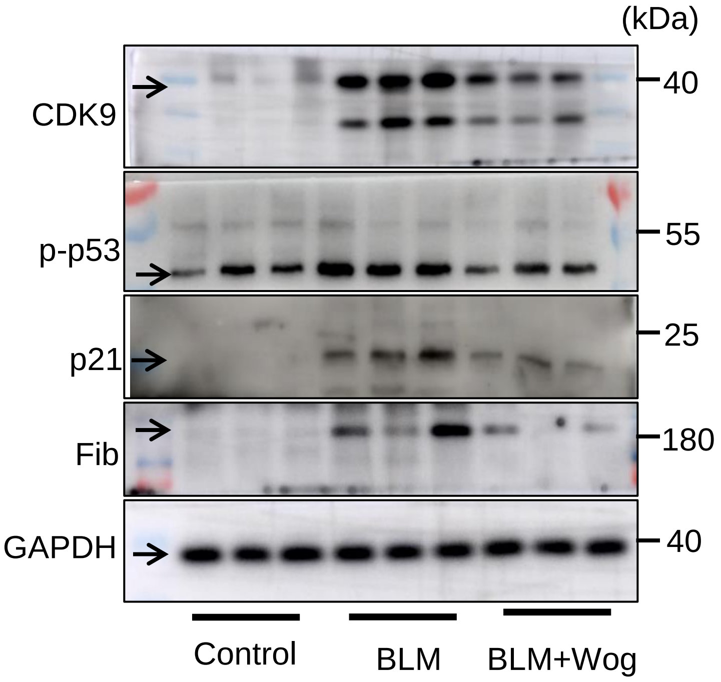

Supplement: Supplementary file 3 [file DataSheet1.PDF]
